# The NF-κB/miR-488/ERBB2 axis modulates pancreatic cancer cell malignancy and tumor growth through cell cycle signaling

**DOI:** 10.1080/15384047.2022.2054257

**Published:** 2022-03-28

**Authors:** Duo Han, Shaihong Zhu, Xia Li, Zhiqiang Li, Hui Huang, Wenzhe Gao, Yunfei Liu, Hongwei Zhu, Xiao Yu

**Affiliations:** a Department of Hepatopancreatobiliary Surgery, The Third Xiangya Hospital, Central South University, Changsha, Hunan Province, China; b Department of Gastrointestinal Surgery, The Third Xiangya Hospital, Central South University, Changsha, Hunan Province, China; c Department of Endocrinology, The Third Xiangya Hospital, Central South University, Changsha, Hunan Province, China

**Keywords:** Pancreatic cancer, miR-488, ERBB2 (receptor tyrosine-protein kinase2), NF-κB, cell cycle signaling

## Abstract

Pancreatic cancer is one of the malignancies having the poorest prognosis due to late diagnoses and lack of efficient treatment regimens. The identification of potential miRNA-targeted gene axes could act as targets for developing novel treatment strategies. Herein, it was assessed that miR-488 expression was markedly downregulated within pancreatic carcinoma. Higher expression of miR-488 was shown to be linked to better prognosis rates of pancreatic carcinoma as per online data. Within two pancreatic tumor cells, MIA PaCa-2 and PANC-1, miR-488 overexpression significantly suppressed malignant cytological behavior by inhibiting cell viability, enhancing cell apoptosis, and inducing cell cycle G2/M-phase arrest. Moreover, miR-488 overexpression also decreased the protein levels of cell cycle regulators, including cyclin A, cyclin B, CDK1, and CDK2. miR-488 directly targets ERBB2 (receptor tyrosine-protein kinase2) to suppress the expression of ERBB2 by targeting its 3ʹUTR. ERBB2 knockdown in MIA PaCa-2 and PANC-1 cell lines suppressed, but miR-488 inhibition enhanced the cancer cell biological malignant behavior; the effects of miR-488 inhibition on pancreatic cancer cells were significantly reversed by ERBB2 knockdown. NF-κB suppressed the expression of miR-488 transcriptionally via targeting its promoter region, consequentially repressing the tumor-suppressive effects of miR-488 upon pancreatic tumor cells. Thus, an NF-κB/miR-488/ERBB2 axis modulating pancreatic cancer cell malignancy and tumor growth through cell cycle signaling was conclusively demonstrated.

## Introduction

Pancreatic cancer is one of the most common malignancies with the poorest prognosis due to delayed diagnosis and lack of effective treatment regimens.^1,2^ Few drugs have been developed to treat pancreatic cancer; however, they have not been satisfactorily conclusive.^3^

MicroRNAs (miRNAs) are aclass of small, endogenous RNAs of 17–25 nucleotides in length,^4^ which post-transcriptionally modulate gene expression.^4–7^ miRNAs often serve as negative regulatory factors of gene expression and provide anew direction in the study of pathological mechanisms and apossible avenue for developing treatment interventions. As recently reported, in comparison with mRNA expression profiles, the miRNA expression pattern is considered aricher source of tumor pathology information.^8^ Moreover, miRNA expression patterns can differ according to cancer and tissue.^8–11^ Consistent with their specific expression patterns, miRNA deregulation in cancers often ensues deregulated cell fate, potentially contributing to tumor invasion, angiogenesis, and metastasis.^12–14^ Many efforts and resources have been invested in miRNA profiling within pancreatic carcinoma. Bloomston etal.^15^ found 11 differentially expressed miRNAs that could differentiate pancreatic carcinoma from normal pancreas and chronic pancreatitis. Another team reviewed previous studies on miRNAs’ functions in cancers and chose atotal of 95 miRNAs for further expression analysis because of their roles in carcinogenesis, cell development, and cell apoptosis and 8 miRNAs, including miR-196a, miR-190, miR-186, miR-221, miR-222, miR-200b, miR-15b, and miR-95, were finally found to be dramatically increased in most pancreatic tumor tissue samples and cells.^16^ Based on these previous findings, the identification of miRNAs potentially correlated with pancreatic cancer carcinogenesis and investigating their specific roles and underlying mechanisms might shed some light on developing novel pancreatic cancer treatment regimens.

With the development of genomic technologies, various teams have been working on identifying pancreatic cancer biomarkers, which could contribute to classifying disease development, treatment response, and potential novel drug targets for cancer therapies; during the past years, no less than 12 molecular pathways were found to play arole in pancreatic cancer carcinogenesis.^17^ Abnormal signaling activities of growth factors and their receptors, transcription factors, and proteins regulating the cell cycle have been getting more involved in the incidence and proliferation of pancreatic cancer; one or several of these molecules may be part of asignal network dysfunction.^18^ More importantly, several miRNAs have been considered as novel pancreatic cancer therapeutics through the regulation of critical molecular pathways; therefore, in further studies, key regulatory factors of pancreatic tumorigenesis should be identified by the integration of deregulated genes and miRNAs into molecular networks. Binding to these key regulatory factors could potentially result in the devising of novel pancreatic carcinoma therapies

During the past recent years, an increasing amount of evidence has been accumulated proving the involvement of the transcription factor NF-κB (Nuclear factor-kappa B), one of the most common molecular changes within pancreatic carcinoma, in the oncogenesis of the pancreas. The inhibition of constitutive NF-κB activation can curb tumor growth and metastasis and sensitize pancreatic tumor cells to the apoptosis induced by the anti-cancer agent.^19^ Its role in malignant transformation, differentiation, cell cycle, and apoptosis have made NF-κB an interesting target for clinical intervention.^20^ Interestingly, several miRNAs, including miR-155,^21,22^ miR-223,^23^ miR-222,^24^ miR-21,^25^ and miR-1908,^26^ have been reported to contain an NF-κB site through which NF-κB transcriptionally regulates their expression. Areasonable hypothesis based on previous findings would be that NF-κB might form regulatory axes with miRNAs and their targets to modulate pancreatic cancer carcinogenesis. Therefore, these possible axes might be potential targets for the development of novel treatment strategies.

The differentially expressed miRNAs between pancreatic cancer and normal control tissue samples were analyzed according to GSE32678; significantly differentially expressed miRNAs were applied for survival analysis, and miR-488 was selected. The specific roles of miR-488 in pancreatic tumor cell phenotypes and pancreatic tumor growth within nude mice were examined. Regarding the downstream mechanism, pancreatic cancer-related pathways were first analyzed based on the data from the TCGA-PAAD database; the miR-488 targets were subsequently with the use of miRDIP, the predicted genes were then applied for the KEGG (Kyoto Encyclopedia of Genes and Genomes) signaling annotation and the GSEA (Gene Set Enrichment Analysis), and these genes were found to be enriched in cell cycle-related pathways. ERBB2 (also known as HER-2/neu) was therefore predicted, akey regulator of the cell cycle, as atarget of miR-488 and was selected for further laboratory analysis. The putative binding of miR-488 to ERBB2 was validated. The specific *invitro* effects of ERBB2 and the dynamic effects of miR-488 and ERBB2 on pancreatic cancer cell phenotypes were examined. Finally, the predicted binding between NF-κB and miR-488 was determined, and the dynamic effects of NF-κB inhibitor and miR-488 upon ERBB2 expression and pancreatic cancer cell phenotype were examined. Anovel NF-κB/miR-488/ERBB2 axis that might affect pancreatic cancer development through the modulation of the cancer cell cycle was conclusively identified.

## Materials and methods

### Clinical sampling

A total of 16 paired pancreatic cancer tissues and adjacent non-cancerous tissues were harvested from patients diagnosed with pancreatic cancer and underwent surgical resection at The Third Xiangya Hospital of Central South University. The patients all signed acomprehensive consent form. All samples were formalin-fixed and paraffin-embedded. All of the experimental procedures were performed after gaining approval from the Ethics Committee of The Third Xiangya Hospital of Central South University.

### Cell lines

Pancreatic cancer cell-line PANC-1 was procured from ATCC (CRL-1469; Manassas, VA, USA) and cultured in Dulbecco’s Modified Eagle’s Medium (30–2002, ATCC) supplemented with 10% FBS (Sigma, St.Louis, MI, USA). MIA PaCa-2 was obtained from ATCC (CRM-CRL-1420) and cultured in Dulbecco’s Modified Eagle’s Medium (30–2002, ATCC) supplemented with 10% FBS. AsPC-1 was obtained from ATCC (CRL-1682), and cultured in an RPMI-1640 medium (30–2001, ATCC) supplemented with 10% FBS (Sigma). Capan-1 was obtained from ATCC (HTB-79), and cultured in an Iscove’s Modified Dulbecco’s Medium (30–2005, ATCC) supplemented with 20% FBS (Sigma).

A human pancreatic nestin-expressing cell line,^27^ hTERT-HPNE, was obtained from ATCC and cultured in amixture of 75% DMEM without glucose (Cat#. D-5030; Sigma, with additional 2 mM L-glutamine and 1.5 g/L sodium bicarbonate) and 25% Medium M3 Base (Cat# M300F-500; Incell Corp., San Antonio, TX, USA) supplemented with 10% FBS (Sigma). All the cells were cultured at 37°C in 5% CO_2_.

### Bio information analysis

Regarding the selection of NF-κB (RELA) expression-related miRNAs in TCGA-PAAD, the online tool Linkedomics (http://linkedomics.org/admin.php) was used. Person’s correlation coefficient analysis was used as astatistical method. Forty-four miRNAs whose expression are negative related with NFκB were selected (FDR < 0.01, P< .01, r< -0.35). Next, online tool KMPLOT (https://kmplot.com/analysis/) was used to analyze the overall survival of the 44 miRNAs in TCGA-PAAD and selected 20 miRNAs (Hazard Ratio < 1, logrank P< .05). Among the 20 miRNAs, the Tstage associated miRNAs selection used Linkedomics to analyze the TCGA-PAAD expression and clinical data. The statistical method used was Jonckheere’s trend test.

Concerning the selection of different miRNAs in pancreatic cancer tissue and normal pancreas tissues, the Gene Expression Omnibus (GEO) dataset GSE32678 (contained 25 human PDAC tumors and 7 nonmalignant pancreas samples) was downloaded using Rlanguage GEOquery package and the differential expression miRNAs were analyzed by Limma package (p < .05, ∣log2FC∣>1).

Regarding overall survival analysis of miR-488 in TCGA-PAAD, KMPLOT with Kaplan-meier and log rank test and Linkedomics with Cox regression test were used. Linkedomics was used for the analysis of miR-488 expression in different TNM stages.

The online tool MIRDIP (https://ophid.utoronto.ca/mirDIP/) was applied for miR-488 targeted genes prediction. These potential targeted genes were applied for KEGG signaling annotation using Rlanguage clusterprofiler package.

The genes having acorrelated expression with mir-488 expression in GSE32688 (contained 25 human PDAC tumors and 7 nonmalignant pancreas samples) and TCGA-PAAD (from linkedomics) were analyzed by Pearson’s correlation coefficient analysis (R language Psych package) and applied for Gene Set Enrichment Analysis (GSEA) using Rlanguage clusterprofiler package.

### Cell transfection

ERBB2 knockdown was generated in target cells through the transfection of si-ERBB2 synthesized by GenePharma (Shanghai, China). Ascramble sequence (si-NC) was used as anegative control. miR-488 overexpression or inhibition was generated in target cells by the transfection of miR-488 mimics or inhibitors synthesized by GenePharma. Regarding NFκB1 overexpression, the full-length human RELA cDNA was cloned in pcDNA3.1 expression vector using ahomologous recombination method. The empty vector pcDNA3.1 was used as anegative control. For cell transfection, 1 × 10^5^ cells were seeded in a6-well cell culture plate, 24 h later, cells were transfected with 50 pmol final concentration miR488-5p mimics, miR488-5p inhibitor or si-ERBB2 through 5 μl per well lipofectamine 3000 (Invitrogen). Following incubation with the RNA Lipofectamine 3000 complex for 6 h, cells were further cultured with fresh complete medium for 48 h and harvested for further laboratory analysis. The sequence of siRNAs and primers for plasmid construction are listed in Table S1.

### Polymerase chain reaction (PCR)-based analyses

Total RNA was extracted, processed, and examined for the expression of target lncRNA, mRNA, and miRNA following the aforementioned methods.^28^ The expression levels of lncRNA, mRNA, and miRNA were detected by aSYBR Green PCR Master Mix (Qiagen, Hilden, Germany) using GAPDH (for mRNA examination) or RNU6B (for miRNA examination) as an endogenous control. The data were processed using a2^−ΔΔCT^ method.

### Lentivirus infection

The lentivirus of miR-488 overexpression (lv-miR-488), miR-488 knockdown (lv-sh-miR-488), and ERBB2 overexpression (lv-ERBB2) were purchased from Genechem (Shanghai, China). Lv-NC and lv-sh-NC were used as negative controls for overexpression or knockdown respectively. The primers for lentivirus transfer vector (Plvx-puro or pLVX-shRNA2-puro) construction are listed in Table S1. Regarding lentivirus infection, 1 × 10^5^ /ml cells were seeded in a6-well cell culture plate overnight. Cells were subsequently incubated with 50 μl lentivirus (1 × 10^8^ TU/ml) for 48 h and collected for an *invivo* Tumorigenicity assay.

### Tumorigenicity assay in mice

Four-week nude Balb/c mice were used as models. All animals were randomly assigned into three groups: Ablank group in which mice bearing tumor derived from lv-NC group (negative control) in which mice bearing tumor derived from PANC-1 cells infected with lv-NC, lv-sh-NC, lv-miR-488, lv-sh-miR-488, or lv-ERBB2. Cells were suspended in 200 μL growth medium/Matrigel and hypodermically injected into left axillaries of the mice in different groups. The tumor volume was determined every 7 days. Twenty-eight days after the injection, mice were sacrificed under anesthesia. The length (L) and width (W) of the tumors were measured with calipers to calculate tumor volumes (V =  L × W^2^/2). The tumor weight was equally determined. The protein levels of Ki-67, PCNA, cyclin A, cyclin B, CDK1, and CDK2 in tumor tissues were determined.

### Immunoblotting

Total protein was extracted, resolved on 10% SDS-PAGE, and transferred onto polyvinylidene fluoride (PVDF) membranes. Nonspecific bindings were blocked by incubation with 5% nonfat dry milk in Tris-buffered saline Tween (TBST) for 2 h. The membranes were subsequently probed with the appropriate primary antibodies at 4°C overnight, followed by another incubation with the corresponding secondary antibodies for 2 h at room temperature. The primary antibodies used are as follows: anti-Ki-67 (ab15580, Abcam, Cambridge, MA, USA), anti-PCNA (ab29, Abcam), anti-cyclin A(18202-1-AP; Proteintech, Wuhan, China), anti-cyclin B(ab32053, Abcam), anti-CDK1 (ab18, Abcam), anti-CDK2 (ab32147, Abcam), anti-ERBB2 (ab16901, Abcam) and anti-GAPDH (ab8245, Abcam). The immunoreactive proteins were visualized and examined using an enhanced chemiluminescence reagent (ECL; BeyoECL Star Kit, Beyotime, Shanghai, China).

### Cell viability examined by CCK-8 assay

Cell viability examination was performed using CCK-8 kit (Beyotime, Shanghai, China). Following transfection or treatment, cells were seeded into 96-well plates at adensity of 5 × 10^3^ cells/well. Two hours prior to the examination, 20 μl of CCK-8 solution was added to each well followed by incubation at 37°C. The Optical density (OD) value was determined at the wavelength of 450 nm on amicroplate reader.

### Cell apoptosis and cell cycle examined by flow cytometry

Regarding for cell apoptosis, cells were transfected and digested 48 h later by trypsin without EDTA and collected. After having been re-suspended in 100 μl binding buffer, the cells were added to 5 μl Annexin V-FITC and 5 μl Propidium Iodide (PI) at room temperature in alight-deprived environment for 15 min. The cells were subsequently tested using the fluorescence-activated cell sorting (FACS) Caliber system (BD Immunocytometry Systems, San Jose, CA, USA).

Regarding for cell cycle, cells were transfected and washed, harvested, and stained 48 h later as per the instructions of the Cell Cycle Analysis Kit (Multi Sciences, Hangzhou, China). The cells were then analyzed using the FACS Caliber system. The percentages of the cells in each phase were determined.

### Chromatin immunoprecipitation (CHIP) assay

An EZ-ChIP kit (Millipore) was used to validate NFκB binding to miR-488 promoter by performing ChIP assays.^29^ Cells were seeded in a6cm dish for 24 h, washed with cold PBS, and then added with 2 ml PBS containing protease inhibitor cocktail after formaldehyde treatment. Discard the supernatant after centrifugation and resuspend in SDS lysis buffer. The cell lysate was centrifuged again and the supernatant was collected. Adilution buffer and protein GSepharose were added and the tube was spined at 4°C for 1 h. The supernatant was collected and incubated with 1 μg anti-p65 (#8242, cell signaling technology, USA) or anti-IgG (negative control) overnight at 4°C with rotation. The supernatant antibody complex was subsequently incubated with 60 μl Protein Gagarose at 4°C with rotation for 1 h. The Protein Gagarose-antibody/chromatin complex was collected by centrifugation and eluted by an elution buffer. Collect the supernatant to reverse the DNA-protein crosslinking. Precipitate and purify the DNA, and detect the level of miR-488 promoter by qRT-PCR. The primers are listed in Table S1. The relative promoter abundance was calculated as the ratio of the amplification efficiency of the ChIP sample to that of the nonimmune IgG.

### Luciferase reporter assay

To validate the binding between miR-488 and ERBB2 3ʹUTR, the study cloned the wild-type or mutated ERBB2 3ʹUTR into the downstream of the Renilla psiCHECK2 vector (Promega, Madison, WI, USA), named wt-ERBB2 3ʹUTR or mut-ERBB2 3ʹUTR. PANC-1 cells were subsequently co-transfected with the luciferase reporter vectors and miR-488 mimics/miR-488 inhibitor.

To validate the binding between NF-κB p65 and miR-488 promoter, the DNA fragment of contained predicted binding site or mutant-binding site were cloned into the downstream of the Renilla psiCHECK2 vector, named miR-488-pro-wt, miR-488-pro-mut. PANC-1 cells were then co-transfected with the luciferase reporter vectors and pcDNA3.1-RELA overexpression vector. 48 h later, the luciferase activity was assessed using the Dual-Luciferase Reporter Assay System (Promega).

### Data processing and statistical analysis

The data was analyzed with aGraphPad software. The measurement data were expressed as mean ± standard deviation (SD). Intergroup data comparisons were performed through aStudent’s *t*-test. Among more than two group data comparisons were performed with the ANOVA followed by aTukey post hoc test. *P*< .05 denoted astatistically significant difference.

## Results

### Selection and verification of miRNAs related to pancreatic cancer carcinogenesis

**Figure 1.**
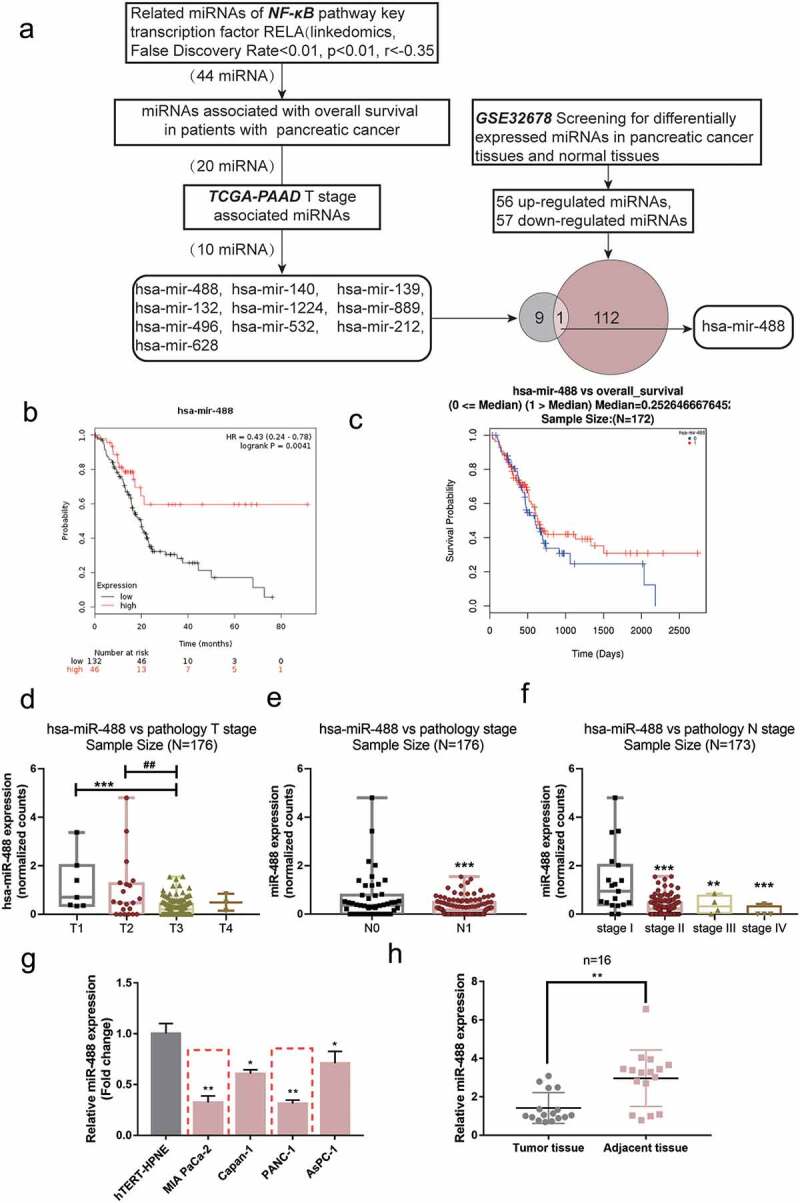
**Selection and verification of miRNAs-related pancreatic cancer carcinoma** (a) Aschematic diagram showing the selecting process of miRNAs-related pancreatic cancer carcinoma. (b) Subjects with pancreatic cancer from the TCGA-PAAD were divided into two groups (high miR-488 expression group and low miR-488 expression group) using the autoselect best value of miR-488 expression as the cutoff. The correlation of miR-488 expression with the survival probability in subjects with pancreatic cancer was analyzed using Kaplan-Meier and log-rank analysis. (c) Subjects with pancreatic cancer from the TCGA database (from linkedomics) were divided into two groups (high miR-488 expression group and low miR-488 expression group) using the median value of miR-488 expression as the cutoff. The correlation of miR-488 expression with the overall survival in subjects with pancreatic cancer was analyzed using aCox-proportional-hazards model (CoxPH). (d) miR-488 expression in Tstages (T1/2/3/4) according to data from TCGA-PAAD database. (e) miR-488 expression in Nstages (n0/1) according to data from TCGA-PAAD database. (f) miR-488 expression in different TNM stages (I/II/III/IV) according to data from TCGA-PAAD database. (g) miR-488 expression was determined in ahuman pancreatic nestin expressing cell line, hTERT-HPNE, and four pancreatic cancer cell lines, PANC-1, BxPC-3, MIA PaCa-2, and Capan-1, by real-time PCR. The red dot line boxes indicated the 2 lowest miR-488 expressed cell lines. n= 3. (h) miR-488 expression was determined in 16 paired pancreatic cancer and adjacent non-cancerous tissues by real-time PCR. n= 16.*P< .05, **P< .01.

To identify miRNAs that might affect pancreatic cancer carcinogenesis, miRNAs related to NF-κB pathway and pancreatic cancer carcinogenesis were selected (Figure1(a)). miRNAs expressively related to RELA were firstly screened, akey transcription factor of the NF-κB pathway; 44 miRNAs were significantly negatively correlated with RELA (False Discovery Rate <0.01, p< .01, r<-0.35) (Figure S1A). Among the 44 miRNAs, 20 miRNAs were associated with the overall survival of TCGA-PAAD using KMPLOT (https://kmplot.com/analysis/) analysis. Next, the online tool linkedomics was used to further select 10 miRNAs associated with the Tstage of pancreatic cancer in TCGA-PAAD. Then, GSE32678 was downloaded and analyzed reporting the differentially expressed genes between pancreatic tumor and normal control tissue samples. According to hierarchical clustering, systematic variation in miRNA expression between cancerous and non-cancerous tissue samples (Figure S1B). One hundred and thirteen differentially expressed miRNAs in total were detected within pancreatic cancer samples, 56 significantly upregulated and 57 downregulated. The distribution of differentially expressed miRNAs between tumor and normal control tissue samples was shown using aVolcano Plot diagram; green and red dots represent markedly decreased and increased miRNAs, respectively (Figure S1C, p< .05, ∣log2FC∣>1). These 113 miRNAs were intersected with the Tstage of TCGA-TAAD associated miRNAs. MiR-488 was subsequently selected. Notably, as predicted by online tool JASPAR (http://jaspar.genereg.net/), miR-488 promoter contains an NF-κB binding site.

As illustrated in Figure1(b), we divided subjects with pancreatic cancer from the TCGA-PAAD database (from KMPLOT) into high and low miR-488 expression groups using the autoselect the best cutoff of miR-488 expression; The Kaplan-Meier and log-rank analyses found that the survival probability increased with an increasing rate of the expression of miR-488. Similarly, subjects with pancreatic cancer from the TCGA-PAAD database (from linkedomics) were allocated into two groups and aCox-proportional-hazards model (CoxPH) showed that higher miR-488 expression was correlated with better overall survival (Figure1(c)). Furthermore, according to data from TCGA-PAAD database (from linkedomics), miR-488 expression was lower in advanced Tstages (Figure1(d)), advanced Nstage (Figure1(e)), and advanced TNM stages (Figure1(f)).

miR-488 expression was subsequently examined in cells and collected tissues. In comparison with that within human pancreatic nestin expressing cells, hTERT-HPNE, the expression of miR-488 showed to be dramatically reduced within four pancreatic tumor cells, PANC-1, BxPC-3, MIA PaCa-2, and Capan-1, more downregulated in MIA PaCa-2 and PANC-1 cells (Figure1(g)). Consistently, the expression of miR-488 showed to be decreased within pancreatic tumor tissue samples than that within adjacent normal control tissue samples (Figure1(h)).

### Specific effects of miR-488 upon pancreatic tumor cell phenotypes

**Figure2.**
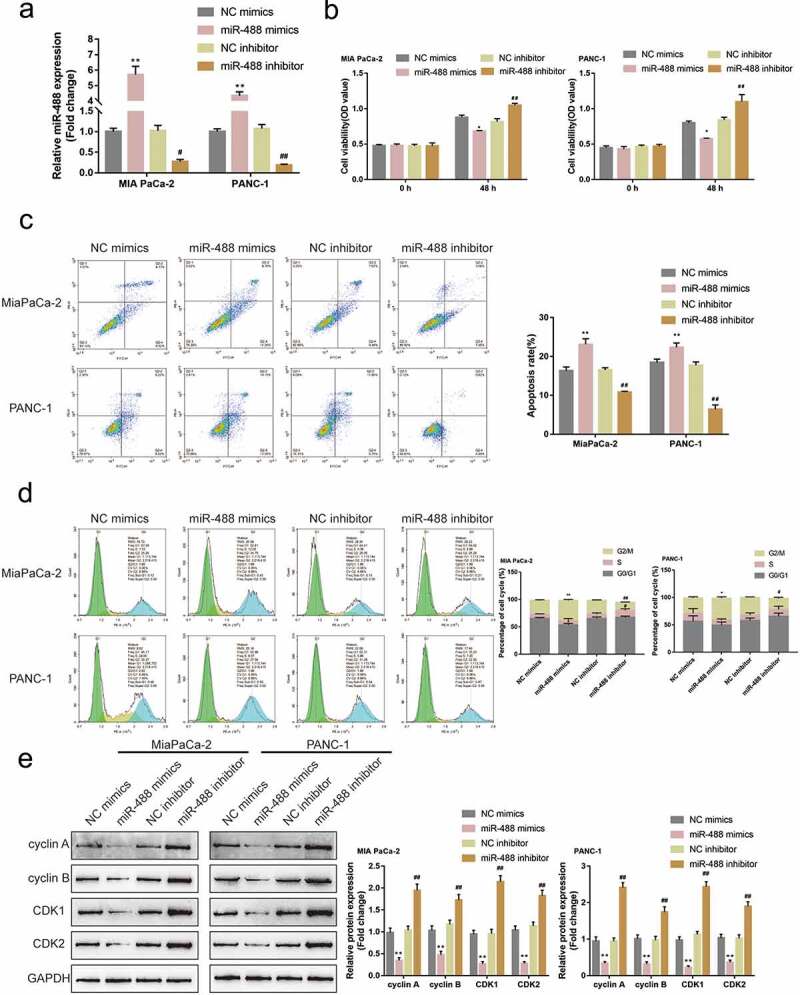
**Specific effects of miR-488 on pancreatic cancer cell phenotypes** (a) miR-488 overexpression or miR-488 inhibition was achieved in MIA PaCa-2 and PANC-1 cells by the transfection of miR-488 mimics or miR-488 inhibitor, as confirmed by real-time PCR (N = 3). Next, MIA PaCa-2 and PANC-1 cells were transfected with miR-488 mimics or miR-488 inhibitor and examined for (b) the cell viability by CCK-8 assay (N = 3); (c) the cell apoptosis by Flow cytometry (N = 3); (d) the cell cycle by Flow cytometry (N = 3); (e) the protein levels of cyclin A, cyclin B, CDK1, and CDK2 by Immunoblotting (N = 3). *P< .05, **P< .01, #*P*< .05, ##*P*< .01.

Firstly, the abnormal downregulation of miR-488 expression was first confirmed within pancreatic carcinoma. Its specific effects on pancreatic cancer cell phenotypes were subsequently investigated. miR-488 mimics/inhibitor were transfected to achieve miR-488 overexpression or miR-488 inhibition within MIA PaCa-2 and PANC-1 cell lines, and areal-time PCR was conducted to verify the transfection efficiency (Figure2(a)). MIA PaCa-2 and PANC-1 cell lines were subsequently selected for further experiments due to the lower miR-488 expression in these two cell lines. miR-488 overexpression remarkably suppressed cell viability, enhanced cell apoptosis, and elicited cell cycle G2/M-phase arrest (Figure2(b-d)); contrastingly, miR-488 inhibition exerted opposite effects (Figure2(b-d)). Consistently, miR-488 overexpression decreased cyclin A, cyclin B, CDK1, and CDK2 protein contents, while miR-488 inhibition increased these proteins (Figure2(e)). In summary, miR-488 could potentially exert atumor-suppressive effect on pancreatic tumor cell lines.

### *Specific effects of miR-488 upon pancreatic tumor growth* invivo

**Figure3.**
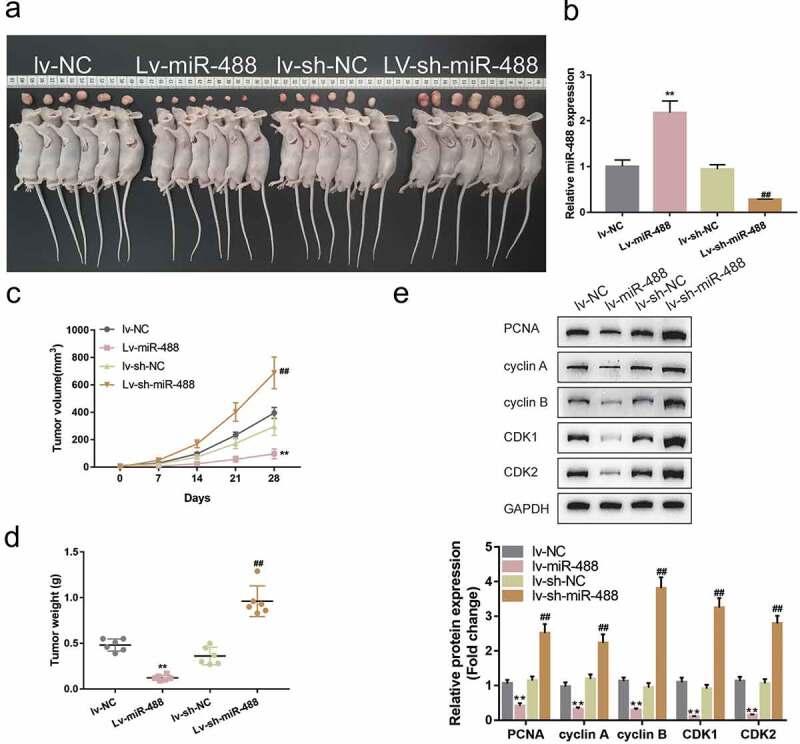
**Specific effects of miR-488 on pancreatic cancer growth *invivo*** mice were randomly assigned into four groups (N = 6). PANC-1 cells, infected with lv-NC, lv-miR-488, lv-sh-NC or lv-sh-miR-488 were hypodermically injected into the left axillaries of mice in different groups. Twenty-eight days after the injection, mice were sacrificed under anesthesia. (a and c) The length (l) and width (w) of the tumors were measured with calipers and the tumor volumes were calculated. n= 6. (b) The infection efficiency of lv-miR-488 or lv-sh-miR-488 was verified by real-time PCR. n= 3. (d) The tumor weight was determined. n= 6. (e-f) The protein levels of Ki-67, PCNA, cyclin A, cyclin B, CDK1, and CDK2 in tumor tissues were determined by Immunoblotting. n= 3. **P< .01 vs. lv-NC; ## *P*< .01 vs. lv-sh-NC.

To further validate the invivo effects of miR-488 upon pancreatic cancer, the nude mouse transplantation tumor experiment was performed. Mice were randomly assigned into four groups and PANC-1 cells, infected with lv-NC, infected with lv-miR-488, infected with lv-sh-NC and infected with lv-sh-miR-488, were hypodermically injected into the left axillaries of mice. Twenty-eight days following the injection, the tumor volumes and the tumor weight were determined. The infection efficiency was verified by areal-time PCR (Figure3(b)). As illustrated in Figure3(a-d), miR-488 overexpression markedly decreased the volume and weight of tumors, while miR-488 knockdown increased tumor growth. Moreover, the protein levels of Ki-67, PCNA, cyclin A, cyclin B, CDK1, and CDK2 were also significantly decreased in tumor tissues in mice bearing miR-488-overexpressing PANC-1 cell-derived tumor (Figure3(e-f)). miR-488 knockdown showed the opposite effects. In summary, miR-488 exerts atumor-suppressive effect on pancreatic carcinoma *invivo*.

### Selection and verification of potential downstream targets of miR-488

**Figure4.**
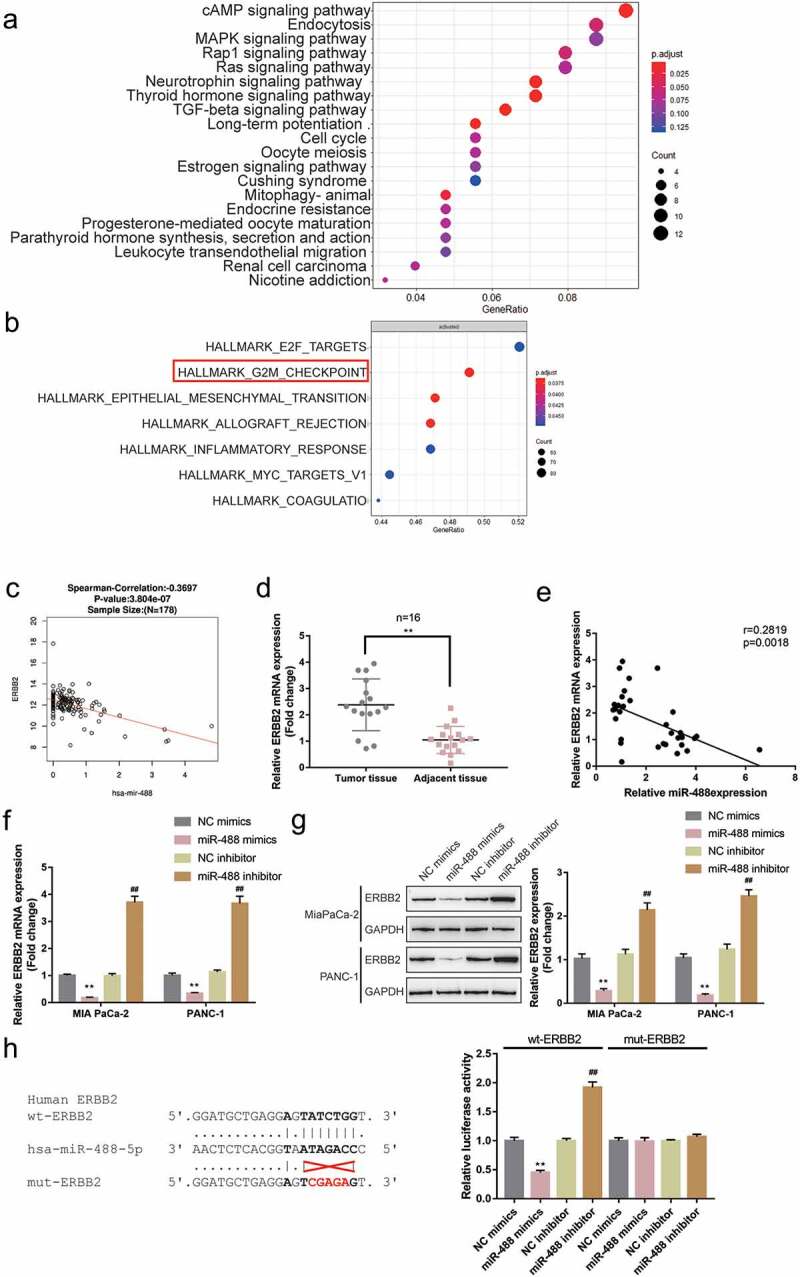
**Selection and verification of potential downstream targets of miR-488** (a) Downstream targets of miR-488 were predicted by miRDIP online tool and predicted genes were applied for the Kyoto Encyclopedia of Genes and Genomes (KEGG) signaling annotation. (b) Genes co-expressed with miR-488 in GSE32688 were selected and applied for the Gene Set Enrichment Analysis (GSEA). (c) The correlation between miR-488 and ERBB2 expression was analyzed using Spearman’s correlation analysis according to data from TCGA-PAAD database (from linkedomics). (d) ERBB2 expression was determined in 16 paired pancreatic cancer and adjacent non-cancerous tissues by real-time PCR. n= 16. (e) The correlation between miR-488 and ERBB2 expression in 16 paired pancreatic cancer and non-cancerous tissues was analyzed using Spearman’s correlation analysis. (f) MIA PaCa-2 and PANC-1 cells were transfected with miR-488 mimics or miR-488 inhibitor and examined for ERBB2 expression by real-time PCR. n= 3. (g) MIA PaCa-2 and PANC-1 cells were transfected with miR-488 mimics or miR-488 inhibitor and examined for ERBB2 protein levels by Immunoblotting. n= 3. (h) Wild- and mutant-type ERBB2 3ʹUTR luciferase reporter vectors were constructed as described and co-transfected in PANC-1 cells with miR-488 mimics or miR-488 inhibitor; the luciferase activity was determined. n= 3. **P< .01 vs. NC mimics, ##*P*< .01 vs. NC inhibitor.

Following the confirmation of the tumor-suppressive effect of miR-488 on pancreatic cancer, the downstream mechanism was further investigated. miR-488 expression-related genes in TCGA-PAAD (from linkedomics) were applied for the Gene Set Enrichment Analysis (GSEA) and the results are depicted in Figure S2A. As illustrated in Figure S2B,C, miR-488 is negatively correlated with pancreatic cancer-related signaling and cell cycle. Mechanistically, miRNAs target mRNAs in their 3’ UTR and lead to gene expression repression, explaining their key roles in cancers;^30^ thus, the online tool miRDIP was employed to predict the possible downstream targets of miR-488. Predicted genes (data not shown) were applied for the KEGG (Kyoto Encyclopedia of Genes and Genomes) signaling annotation. As depicted in Figure4(a), predicted miR-488 downstream targets were significantly enriched in cell cycle factors and were correlated with cell division. Moreover, genes co-expressed with miR-488 in GSE32688 were selected and applied for the GSEA. As depicted in Figure4(b), these genes were significantly enriched in cell cycle regulation (G2M checkpoint). The correlation between miR-488 and these genes was subsequently analyzed (data not shown) and found that miR-488 was significantly correlated with ERBB2 expression according to data from TCGA-PAAD database (Figure4(c)). ERBB2 (also known as CD340 and HER2/neu) is awell-known proto-oncogene that modulates the expression and function of cell cycle regulators.^31^ It was therefore hypothesized that miR-488 might exert its effects through ERBB2.

In pancreatic cancer tissues, ERBB2 expression was significantly upregulated than that within normal control tissues (Figure4(d)). Within tissues, ERBB2 expression was negatively correlated with miR-488 expression (Figure4(e)). Within both MIA PaCa-2 and PANC-1 cell lines, miR-488 overexpression was significantly downregulated, while miR-488 inhibition upregulated ERBB2 expression (Figure4(f)); consistently, miR-488 overexpression was dramatically inhibited in both cell lines, while the inhibition of miR-488 enhanced ERBB2 protein levels (Figure4(g)).

To investigate the predicted binding of miR-488 to ERBB2, aluciferase reporter assay was performed. Two different types of ERBB2 3ʹUTR luciferase reporter vectors were constructed as described, wild-type, and mutant-type, these vectors were subsequently co-transfected in PANC-1 cells with miR-488 mimics/inhibitor. Figure4(h) illustrates that wt-ERBB2 3ʹUTR vector’s luciferase activity was markedly repressed via the overexpression of miR-488 while it was enhanced via the inhibition of miR-488; mutating the putative miR-488 binding site could abolish the changes in the luciferase activity. In summary, miR-488 suppresses the expression of ERBB2 via targeting its 3ʹUTR.

### Specific effects of ERBB2 upon pancreatic tumor cell phenotype

**Figure5.**
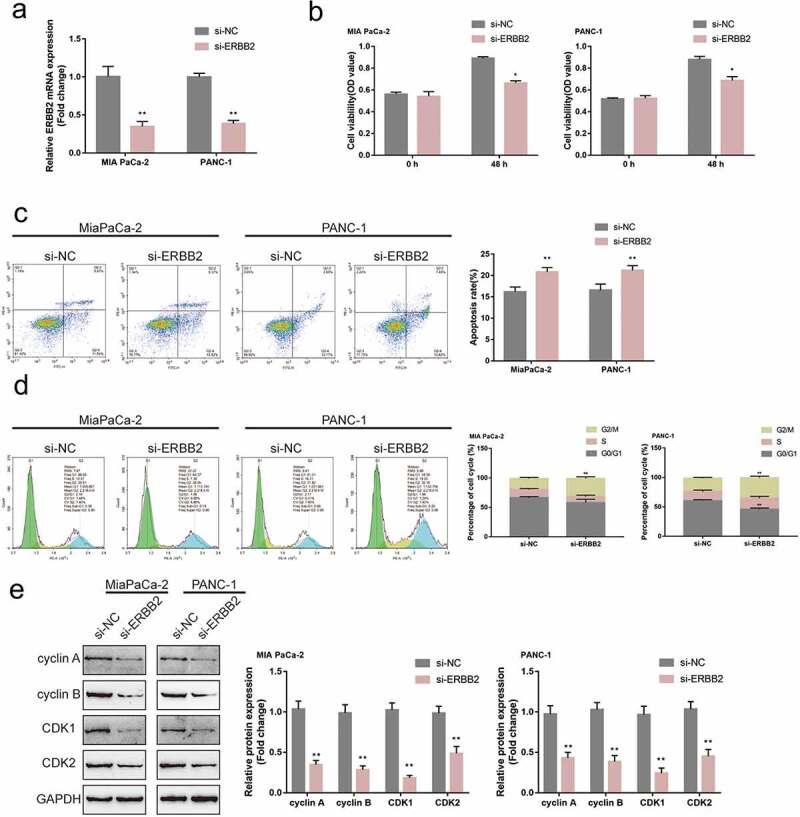
**Specific effects of ERBB2 on pancreatic cancer cell phenotype** (a) ERBB2 knockdown was achieved in MIA PaCa-2 and PANC-1 cells by the transfection of si-ERBB2, as confirmed by real-time PCR (N = 3). Next, MIA PaCa-2 and PANC-1 cells were transfected with si-ERBB2 and examined for (b) the cell viability by CCK-8 assay (N = 3); (c) the cell apoptosis by Flow cytometry (N = 3); (d) the cell cycle by Flow cytometry (N = 3); (e) the protein levels of cyclin A, cyclin B, CDK1, and CDK2 by Immunoblotting (N = 3). *P< .05, **P< .01.

After confirmation of the binding between miR-488 and ERBB2, the specific effects of ERBB2 upon pancreatic tumor cell lines were further investigated. si-ERBB2 was transfected to achieve ERBB2 knockdown within MIA PaCa-2 and PANC-1 cell lines, areal-time PCR was performed to verify the transfection efficiency (Figure5(a)). In MIA PaCa-2 and PANC-1 cell lines, ERBB2 knockdown exerted similar effects as miR-488 overexpression by inhibiting the cell viability, promoting the cell apoptosis, and eliciting cell cycle G2/M-phase arrest (Figure5(b-d)). Consistently, ERBB2 knockdown also decreased cyclin A, cyclin B, CDK1, and CDK2 protein contents in both pancreatic tumor cells (Figure5(e)). In summary, in pancreatic tumor cells, ERBB2 also plays an oncogenic role.

### miR-488 modulates pancreatic cancer cell phenotype through ERBB2

**Figure6.**
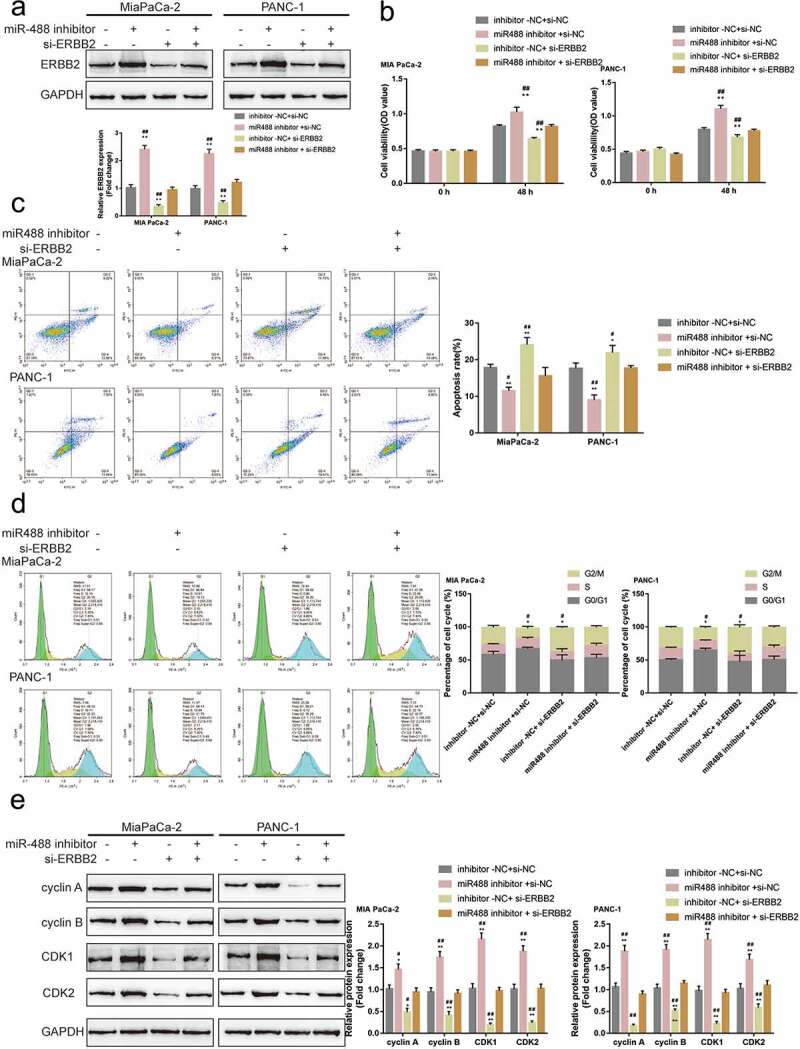
**miR-488 modulates pancreatic cancer cell phenotype through ERBB2** MIA PaCa-2 and PANC-1 cells were co-transfected with miR-488 inhibitor and si-ERBB2 and examined for (a) the protein level of ERBB2 (N = 3); (b) the cell viability by CCK-8 assay (N = 3); (c) the cell apoptosis by Flow cytometry (N = 3); (d) the cell cycle by Flow cytometry (N = 3); (e) the protein levels of cyclin A, cyclin B, CDK1, and CDK2 by Immunoblotting (N = 3). *P< .05, **P< .01, compared to the control group; #*P*< .05, ##*P*< .01, compared to miR-488 inhibitor + si-ERBB2 group.

**Figure7.**
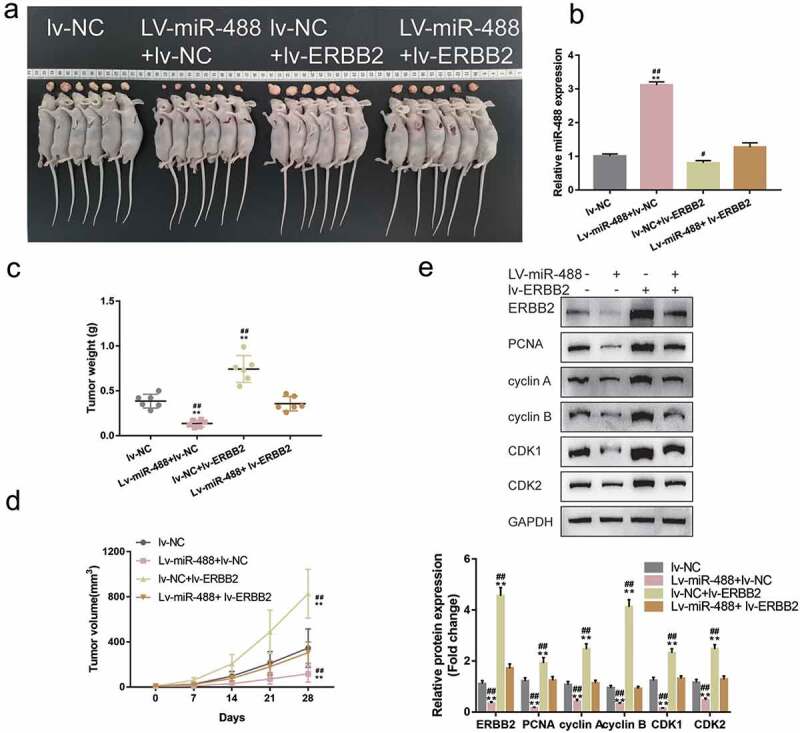
**Specific effects of miR-488 and ERBB2 on pancreatic cancer growth *invivo.*** mice were randomly assigned into four groups (N = 6). PANC-1 cells, infected with lv-NC, lv-miR-488, lv-ERBB2 or lv-miR-488+ lv-ERBB2 were hypodermically injected into the left axillaries of mice in different groups. Twenty-eight days after the injection, mice were sacrificed under anesthesia. (a and c) The length (l) and width (w) of the tumors were measured with calipers and the tumor volumes were calculated (N = 6). (b) The levels of miR-488 in tumor tissues were verified by real-time PCR. (d) The tumor weight was determined (N = 3). (e-f) The protein levels of ERBB2, Ki-67, PCNA, cyclin A, cyclin B, CDK1, and CDK2 in tumor tissues were determined by Immunoblotting (N = 3). **P< .01 vs. lv-NC; ## *P*< .01 vs. lv-miR-488+ lv-ERBB2.

Since miR-488 directly targets ERBB2, the dynamic effects of miR-488 and ERBB2 on pancreatic tumor cell lines were subsequently examined. MIA PaCa-2 and PANC-1 cell lines were co-transfected with miR-488 inhibitor and si-ERBB2 and examined for related indexes. Figure6(a) shows that the inhibition of miR-488 inhibition was upregulated, while ERBB2 knockdown downregulated the protein level of ERBB2; the effects of miR-488 inhibition on ERBB2 protein level were significantly reversed by ERBB2 knockdown. Regarding pancreatic cancer cell phenotype, miR-488 inhibition promoted the cell viability, repressed the cell apoptosis, and caused no arrest in the cell cycle (Figure6(b-d)); contrariwise, ERBB2 knockdown inhibited the cell viability, enhanced the cell apoptosis, and elicited cell cycle G2/M-phase arrest (Figure6(b-d)). More importantly, ERBB2 knockdown significantly attenuated the effects of miR-488 inhibition (Figure6(b-d)). Concerning the cell cycle-related factors, miR-488 inhibition was upregulated, while ERBB2 knockdown downregulated cyclin A, cyclin B, CDK1, and CDK2 protein contents; the effects of miR-488 inhibition were significantly reversed by ERBB2 knockdown (Figure6(e)). Moreover, to further validate the *invivo* effects of miR-488/ERBB2 upon pancreatic cancer, the nude mouse transplantation tumor experiment was performed. The infection efficiency of miR-488 or ERBB2 overexpression was verified by areal-time PCR or western-blot (Figure7(b,e)). ERBB2 overexpression increased the tumor growth and increased the protein levels of Ki-67, PCNA, cyclin A, cyclin B, CDK1, and CDK2 in tumor tissues. miR-488 overexpression significantly reversed the effect of ERBB2 on tumor growth and cell cycle-related proteins expression (Figure7(a,c-e)). miR-488/ERBB2 axis has therefore been conclusively shown to be involved in pancreatic cancer progress.

### NF-κB transcriptionally inhibits miR-488 expression to affect its function

**Figure8.**
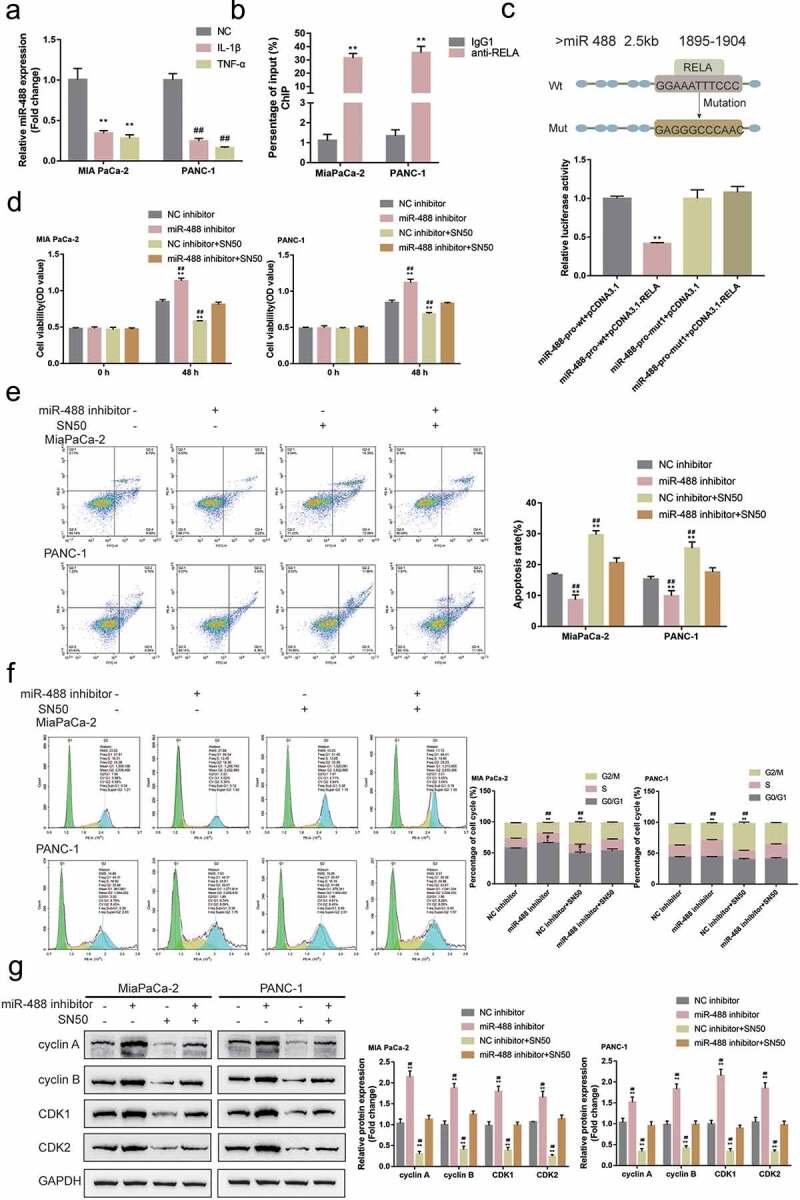
**NF-κB transcriptionally inhibits miR-488 expression to affect its function** (a) MIA PaCa-2 and PANC-1 cells were treated with IL-1β (10 ng/ml) or TNF-α (50 ng/ml) for 24 h and examined for the expression of miR-488 by real-time PCR (N = 3). (b) CHIP assay was performed to analyze the miR-488 promoter fragment levels in NFκB (RELA) immunoprecipitate (N = 3). (c) Wild- and mutant-type miR-488 luciferase reporter vectors were constructed and co-transfected in PANC-1 cells with pcDNA3.1/NF-κB; the luciferase activity was determined. Next, MIA PaCa-2 and PANC-1 cells were transfected with miR-488 inhibitor in the presence or absence of NF-κB inhibitor SN50 (18 μM) and examined for (d) the cell viability by CCK-8 assay (N = 3); (e) the cell apoptosis by Flow cytometry (N = 3); (f) the cell cycle by Flow cytometry (N = 3); (g) the protein levels of cyclin A, cyclin B, CDK1, and CDK2 by Immunoblotting (N = 3). *P< .05, **P< .01, compared to the control group; #*P*< .05, ##*P*< .01, compared to miR-488 inhibitor + SN50 group.

The transcription factor NF-κB in the oncogenesis of the pancreas has been accumulated over along period due to its effect on malignant cell transformation, differentiation, cell cycle, and apoptosis.^19,20^ As analyzed by JASPAR (http://jaspar.genereg.net/), there is one NF-κB (RELA) binding site within the miR-488 promoter region (score >10); thus, the investigation regarding whether NF-κB could transcriptionally regulate miR-488 expression was sustained. MIA PaCa-2 and PANC-1 cell lines were treated with IL-1β or TNF-α for 24 h, miR-488 expression was determined; Figure7(a) shows that single IL-1β or TNF-α treatment significantly inhibited miR-488 expression. ACHIP assay was subsequently performed to determine whether NFκB bind to the miR-488 promoter. As illustrated by Figure8(b), in MiaPaCa-2 and PANC-1 cells, the level of miR-488 promoter fragment containing the binding site was significantly higher in the NFκB immunoprecipitate, when compared to the IgG group. Aluciferase reporter assay was conducted to investigate the putative binding of NF-κB to miR-488 promoter region. Two different types of miR-488 luciferase reporter vectors were co-transfected, wild-type, and mutant-type, these vectors in PANC-1 cells were co-transfected with pcDNA3.1/NF-κB; Within the putative NF-κB binding site of mutant-type reporter vectors, several bases have been mutated to remove the complementary (Figure8(c)). Figure8(c) shows that the wild-type reporter vector’s luciferase activity was significantly inhibited by pcDNA3.1-NF-κB; the mutation of the putative NF-κB binding sites could abolish the alterations in the luciferase activity (Figure8(c)). NF-κB conclusively inhibits the expression of miR-488 via targeting its promoter region.

MIA PaCa-2 and PANC-1 cell lines were transfected with miR-488 inhibitor with or without NF-κB inhibitor SN50 and examined for related indexes. Figure8(d-f) showed that miR-488 inhibition enhanced, while SN50 treatment attenuated pancreatic tumor cell aggressiveness; the effects of SN50 treatment were significantly reversed by miR-488 inhibition. Regarding the protein levels of cell cycle regulators, miR-488 inhibition increased, while SN50 treatment decreased cyclin A, cyclin B, CDK1, and CDK2 protein contents; similarly, miR-488 inhibition significantly attenuated the effects of SN50 treatment (Figure8(g)). In summary, NF-κB inhibits miR-488 expression transcriptionally to affect miR-488 functions on pancreatic cancer cells.

## Discussion

Herein, it was concluded that miR-488 expression showed to be markedly downregulated within pancreatic carcinoma and that higher expression of miR-488 showed to be linked to abetter prognosis of pancreatic carcinoma patients according to online data. Within two pancreatic tumor cells, MIA PaCa-2 and PANC-1, miR-488 overexpression markedly suppressed the cancer cell biological malignant behavior through the inhibition of cell viability, enhancing cell apoptosis, and inducing cell cycle G2/M-phase arrest. Furthermore, miR-488 overexpression also decreased the protein levels of cell cycle regulators, including cyclin A, cyclin B, CDK1, and CDK2. miR-488 directly targets ERBB2; miR-488 suppresses the expression of ERBB2 via targeting its 3ʹUTR. ERBB2 knockdown in MIA PaCa-2 and PANC-1 cell lines suppressed, however, miR-488 inhibition enhanced the cancer cell cytological malignant behavior; the effects of miR-488 inhibition on pancreatic cancer cells were significantly reversed by ERBB2 knockdown. NF-κB suppressed the expression of miR-488 transcriptionally via targeting its promoter region, thus repressing the tumor-suppressive effects of miR-488 upon pancreatic tumor cells.

With the significant advent of pancreatic carcinoma cytology in recent years, it has been found that the miRNA families have exerted asignificant effect on the modulation of tumor response. As reported, the miR-200 family was remarkably decreased within gemcitabine resistant pancreatic tumor cells and the upregulation of the miR-200 family could reverse the EMT (epithelial–mesenchymal transition).^32^ miR-34 is involved in cancer stem cell maintenance and survival through the regulation of Notch pathway proteins and Bcl-2. The upregulation of miR-34 might partially reverse the tumor-suppressive effects of p53 within p53-deficient human pancreatic tumor cell lines through inhibiting the growth and invasion of clonogenic cells, inducing cell apoptosis and cell cycle arrest in G1 and G2/M phases, and sensitizing the cells to chemotherapy and radiotherapy.^33^ Coupled with these reported miRNAs, aconsequential number of deregulated miRNAs in pancreatic cancer,^15–17^ are thought to be involved in pancreatic cancer carcinoma. It was therefore concluded that miR-488 was significantly reduced within both pancreatic tumor tissue samples and cells using bioinformatics and experimental analyses. As reported, the expression of miR-488 was abnormally downregulated within various cancers, such as gastric carcinoma,^34^ malignant melanoma,^35^ ovarian cancer,^36^ tongue squamous carcinoma,^37^ renal cell carcinoma,^38^ as well as pancreatic ductal adenocarcinoma.^39^ More importantly, miR-488 expression was more downregulated in subjects in advanced TNM stages, suggesting that miR-488 potentially participated in pancreatic cancer progression.

Consistent with its specific expression pattern, miR-488 exerts atumor-suppressive effect on numerous cancers by affecting almost every aspect of cancer malignancy. By targeting activating transcription factor 3 (ATF3), miR-488 inhibits the EMT of tongue squamous carcinoma cells, therefore inhibiting cancer cell invasion.^37^ By targeting claudin-1 and the MAPK signaling pathway, miR-488 inhibited colorectal cancer SW480 cell viability, invasion, and migratory ability and enhanced cell apoptosis.^40^ In pancreatic ductal adenocarcinoma, miR-488 targeted JAK1 and JAK1/STAT3 signaling to inhibit pancreatic ductal adenocarcinoma metastasis.^39^ Herein, according to the bioinformatics analysis, the predicted targets of miR-488 were significantly enriched in the cell cycle-related signaling. Later, experimental analysis confirmed that miR-488 overexpression inhibited pancreatic cancer cell viability, enhanced apoptosis, and elicited cell cycle G2/M-phase arrest. Consistently, miR-488 overexpression decreased the protein levels of cell cycle regulators, cyclin A, cyclin B, CDK1, and CDK2. *In vivo*, miR-488 overexpression inhibited tumor growth in nude mice transplantation model accompanied with decreased protein levels of cell cycle regulators. In summary, miR-488 may play atumor-suppressive role through the modulation of pancreatic cancer cell cycle.

The central components of the cell-cycle control system are cyclin-dependent protein kinases (CDKs), where its activity depends on association with regulatory subunits called cyclins. CDK1, CDK2, CDK3, CDK4, and CDK6, belonging to the serine/threonine protein kinase family (CDKs), appear to drive cell cycle progression.^41^ Two key players at the G1-S checkpoints, cyclin D-CDK4/6 and cyclin E-CDK2 complexes, drive phosphorylation of the retinoblastoma protein (RB), leading to the initiation of DNA replication and mitosis. Cyclin Ais associated with CDK2 and plays arole at both the cell cycle G1-S and G2-M checkpoints.^42^ Overexpression of G1-S cyclins and CDKs in the process of PC carcinogenesis are well defined.^43^ Cyclin D3 and cyclin Awere found to be overexpressed within 90 to 100% of high grade pancreatic intraepithelial neoplasia and pancreatic carcinoma.^43^ Besides, CDK2 and CDK4 were overexpressed within early-stage pancreatic intraepithelial neoplasias, and gradually elevated to 60 to 75% within pancreatic carcinoma.^44^ In pancreatic cancer, ERBB2 (HER2) and EGF-R bind to their ligands, including EGF and TGF-α, to activate the RAS signaling pathway,^45^ which play akey role in the activation of cyclin D1 through assembly with CDKs. This directly leads to the phosphorylation and inactivation of the growth-suppressive retinoblastoma protein (Rb) in cell cycle G1 phase, therefore resulting in deregulated cell proliferation.^46^ Herein, both the bioinformatics and experimental analyses have reported that miR-488 directly targets ERBB2. Similar to miR-488 overexpression, ERBB2 knockdown in pancreatic cancer cells attenuated the cancer cell malignancy by inhibiting the cell viability, promoting cell apoptosis, and eliciting cell cycle G2/M-phase arrest. Consistent with previous studies, ERBB2 knockdown invitro decreased cyclin A, cyclin B, CDK1, and CDK2 protein contents. More importantly, ERBB2 knockdown significantly reversed the roles of miR-488 inhibition in pancreatic tumor cell lines, indicating that miR-488 exerts atumor-suppressive effect on pancreatic carcinoma through targeting ERBB2 to modulate the cancer cell cycle.

Since miR-488 was abnormally downregulated within pancreatic carcinoma, the investigation regarding the potential mechanism was sustained. As reported, the activity of NF-κB is constitutively activated within approximately 70% of pancreatic tumors.^47^ Although NF-κB is found to transcriptionally modulate miRNA expression via targeting miRNA promoter region. This modulation, therefore, affects the carcinogenesis of several cancers. However, comparable studies are limited in pancreatic cancer. In this study, JASPAR and experimental analyses both indicated two possible NF-κB-binding sites within the promoter region of miR-488. Through binding to the promoter region, NF-κB inhibits miR-488 expression, therefore enhancing the malignant traits of pancreatic cancer cells; upon NF-κB inhibitor treatment, cancer cell malignancy was reduced. These data conclusively prove that NF-κB transcriptionally inhibits miR-488 expression to affect downstream ERBB2 expression, therefore affecting pancreatic cancer cell cycle, cell viability, and cell apoptosis.

A NF-κB/miR-488/ERBB2 axis modulating pancreatic cancer cell malignancy and tumor growth through cell cycle signaling is therefore conclusively demonstrated.

## Supplementary Material

Supplemental Material

## References

[1] Zhao C, Gao F, Li Q, Liu Q, Lin X. The distributional characteristic and growing trend of pancreatic cancer in China. Pancreas. 2019;48:309–314. doi:10.1097/MPA.0000000000001222. 30855427

[2] Aier I, Semwal R, Sharma A, Varadwaj PK. Asystematic assessment of statistics, risk factors, and underlying features involved in pancreatic cancer. Cancer Epidemiol. 2019;58:104–110. doi:10.1016/j.canep.2018.12.001. 30537645

[3] Matera R, Saif MW. New therapeutic directions for advanced pancreatic cancer: cell cycle inhibitors, stromal modifiers and conjugated therapies. Expert Opin Emerg Drugs. 2017;22:223–233. doi:10.1080/14728214.2017.1362388. 28783977

[4] Lee RC, Feinbaum RL, Ambros V. The C.elegans heterochronic gene lin-4 encodes small RNAs with antisense complementarity to lin-14. Cell. 1993;75:843–854. doi:10.1016/0092-8674(93)90529-Y. 8252621

[5] Pasquinelli AE, Reinhart BJ, Slack F, Martindale MQ, Kuroda MI, Maller B, Hayward DC, Ball EE, Degnan B, Müller P, etal. Conservation of the sequence and temporal expression of let-7 heterochronic regulatory RNA. Nature. 2000;408:86–89. doi:10.1038/35040556. 11081512

[6] Ambros V. The functions of animal microRNAs. Nature. 2004;431:350–355. doi:10.1038/nature02871. 15372042

[7] Bartel DP. MicroRNAs: genomics, biogenesis, mechanism, and function. Cell. 2004;116:281–297. doi:10.1016/S0092-8674(04)00045-5. 14744438

[8] Lu J, Getz G, Miska EA, Alvarez-Saavedra E, Lamb J, Peck D, Sweet-Cordero A, Ebert BL, Mak RH, Ferrando AA, etal. MicroRNA expression profiles classify human cancers. Nature. 2005;435:834–838. doi:10.1038/nature03702. 15944708

[9] Yanaihara N, Caplen N, Bowman E, Seike M, Kumamoto K, Yi M, Stephens RM, Okamoto A, Yokota J, Tanaka T, etal. Unique microRNA molecular profiles in lung cancer diagnosis and prognosis. Cancer Cell. 2006;9:189–198. doi:10.1016/j.ccr.2006.01.025. 16530703

[10] He H, Jazdzewski K, Li W, Liyanarachchi S, Nagy R, Volinia S, Calin GA, Liu C-G, Franssila K, Suster S, etal. The role of microRNA genes in papillary thyroid carcinoma. Proc Natl Acad Sci USA. 2005;102:19075–19080. doi:10.1073/pnas.0509603102. 16365291 PMC1323209

[11] Iorio MV, Ferracin M, Liu CG, Veronese A, Spizzo R, Sabbioni S, Magri E, Pedriali M, Fabbri M, Campiglio M, etal. MicroRNA gene expression deregulation in human breast cancer. Cancer Res. 2005;65:7065–7070. doi:10.1158/0008-5472.CAN-05-1783. 16103053

[12] Roldo C, Missiaglia E, Hagan JP, Falconi M, Capelli P, Bersani S, Calin GA, Volinia S, Liu C-G, Scarpa A, etal. MicroRNA expression abnormalities in pancreatic endocrine and acinar tumors are associated with distinctive pathologic features and clinical behavior. JClin Oncol. 2006;24:4677–4684. doi:10.1200/JCO.2005.05.5194. 16966691

[13] Calin GA, Croce CM. MicroRNA signatures in human cancers. Nat Rev Cancer. 2006;6:857–866. doi:10.1038/nrc1997. 17060945

[14] Garzon R, Fabbri M, Cimmino A, Calin GA, Croce CM. MicroRNA expression and function in cancer. Trends Mol Med. 2006;12:580–587. doi:10.1016/j.molmed.2006.10.006. 17071139

[15] Bloomston M, Frankel WL, Petrocca F, Volinia S, Alder H, Hagan JP, Liu C-G, Bhatt D, Taccioli C, Croce CM, etal. MicroRNA expression patterns to differentiate pancreatic adenocarcinoma from normal pancreas and chronic pancreatitis. JAMA. 2007;297:1901–1908. doi:10.1001/jama.297.17.1901. 17473300

[16] Zhang Y, Li M, Wang H, Fisher WE, Lin PH, Yao Q, Chen C. Profiling of 95 microRNAs in pancreatic cancer cell lines and surgical specimens by real-time PCR analysis. World JSurg. 2009;33:698–709. doi:10.1007/s00268-008-9833-0. 19030927 PMC2933040

[17] Lopez-Casas PP, Lopez-Fernandez LA. Gene-expression profiling in pancreatic cancer. Expert Rev Mol Diagn. 2010;10:591–601. doi:10.1586/erm.10.43. 20629509

[18] Reddy SA. Signaling pathways in pancreatic cancer. Cancer J. 2001;7:274–286. 11561604

[19] Sclabas GM, Fujioka S, Schmidt C, Evans DB, Chiao PJ. NF-kappaB in pancreatic cancer. IntJGastrointest Cancer. 2003;33:15–26. doi:10.1385/IJGC:33:1:15. 12909735

[20] Algul H, Adler G, Schmid RM. NF-kappaB/Rel transcriptional pathway: implications in pancreatic cancer. IntJGastrointest Cancer. 2002;31:71–78. doi:10.1385/IJGC:31:1-3:71. 12622417

[21] Wang P, Zhu CF, Ma MZ, Chen G, Song M, Zeng ZL, Lu W-H, Yang J, Wen S, Chiao PJ, etal. Micro-RNA-155 is induced by K-Ras oncogenic signal and promotes ROS stress in pancreatic cancer. Oncotarget. 2015;6:21148–21158. doi:10.18632/oncotarget.4125. 26020803 PMC4673256

[22] Gatto G, Rossi A, Rossi D, Kroening S, Bonatti S, Mallardo M. Epstein-Barr virus latent membrane protein 1 trans-activates miR-155 transcription through the NF-kappaB pathway. Nucleic Acids Res. 2008;36:6608–6619. doi:10.1093/nar/gkn666. 18940871 PMC2582607

[23] Kumar V, Palermo R, Talora C, Campese AF, Checquolo S, Bellavia D, Tottone L, Testa G, Miele E, Indraccolo S, etal. Notch and NF-kB signaling pathways regulate miR-223/FBXW7 axis in T-cell acute lymphoblastic leukemia. Leukemia. 2014;28:2324–2335. doi:10.1038/leu.2014.133. 24727676

[24] Orecchini E, Doria M, Michienzi A, Giuliani E, Vassena L, Ciafre SA, Farace MG, Galardi S. The HIV-1 Tat protein modulates CD4 expression in human Tcells through the induction of miR-222. RNA Biol. 2014;11:334–338. doi:10.4161/rna.28372. 24717285 PMC4075518

[25] Madhyastha R, Madhyastha H, Pengjam Y, Nakajima Y, Omura S, Maruyama M. NFkappaB activation is essential for miR-21 induction by TGFbeta1 in high glucose conditions. Biochem Biophys Res Commun. 2014;451:615–621. doi:10.1016/j.bbrc.2014.08.035. 25130469

[26] Kuang Q, Li J, You L, Shi C, Ji C, Guo X, Xu M, Ni Y. Identification and characterization of NF-kappaB binding sites in human miR-1908 promoter. Biomed Pharmacother. 2015;74:158–163. doi:10.1016/j.biopha.2015.08.018. 26349979

[27] Cecati M, Giulietti M, Righetti A, Sabanovic B, Piva F. Effects of CXCL12 isoforms in apancreatic pre-tumour cellular model: microarray analysis. World JGastroenterol. 2021;27:1616–1629. doi:10.3748/wjg.v27.i15.1616. 33958847 PMC8058651

[28] Liu Y, Wang Y, He X, Zhang S, Wang K, Wu H, Chen L. LncRNA TINCR/miR-31-5p/C/EBP-alpha feedback loop modulates the adipogenic differentiation process in human adipose tissue-derived mesenchymal stem cells. Stem Cell Res. 2018;32:35–42. doi:10.1016/j.scr.2018.08.016. 30172905

[29] Liu K, Chen W, Lei S, Xiong L, Zhao H, Liang D. Wild-type and mutant p53 differentially modulate miR-124/iASPP feedback following photodynamic therapy in human colon cancer cell line. Cell Death Dis. 2017;8:e3096. doi:10.1038/cddis.2017.477. 29022915 PMC5682646

[30] Palanichamy JK, Rao DS. Rao DS. miRNA dysregulation in cancer: towards amechanistic understanding. Front Genet. 2014;5:54. doi:10.3389/fgene.2014.00054. 24672539 PMC3957189

[31] Busse D, Doughty RS, Arteaga CL. HER-2/neu (erbB-2) and the cell cycle. Semin Oncol. 2000;27:3–8. discussion 92-100. 11236025

[32] Li Y, VandenBoom TG 2nd, Kong D, Wang Z, Ali S, Philip PA, Sarkar FH. Up-regulation of miR-200 and let-7 by natural agents leads to the reversal of epithelial-to-mesenchymal transition in gemcitabine-resistant pancreatic cancer cells. Cancer Res. 2009;69:6704–6712. doi:10.1158/0008-5472.CAN-09-1298. 19654291 PMC2727571

[33] Ji Q, Hao X, Zhang M, Tang W, Yang M, Li L, Xiang D, DeSano JT, Bommer GT, Fan D, etal. MicroRNA miR-34 inhibits human pancreatic cancer tumor-initiating cells. PLoS One. 2009;4:e6816. doi:10.1371/journal.pone.0006816. 19714243 PMC2729376

[34] Zhao Y, Lu G, Ke X, Lu X, Wang X, Li H. miR-488 acts as atumor suppressor gene in gastric cancer. Tumour Biol. 2016;37:8691–8698. doi:10.1007/s13277-015-4645-y. 26738864

[35] Li N, Ma Y, Ma L, Guan Y, Ma L, Yang D. MicroRNA-488-3p sensitizes malignant melanoma cells to cisplatin by targeting PRKDC. Cell Biol Int. 2017;41:622–629. doi:10.1002/cbin.10765. 28328082

[36] Yang Z, Feng Z, Gu J, Li X, Dong Q, Liu K, Li Y, OuYang L. microRNA-488 inhibits chemoresistance of ovarian cancer cells by targeting Six1 and mitochondrial function. Oncotarget. 2017;8:80981–80993. doi:10.18632/oncotarget.20941. 29113360 PMC5655255

[37] Shi B, Yan W, Liu G, Guo Y. MicroRNA-488 inhibits tongue squamous carcinoma cell invasion and EMT by directly targeting ATF3. Cell Mol Biol Lett. 2018;23:28. doi:10.1186/s11658-018-0094-0. 29946339 PMC6006839

[38] Wei X, Yu L, Kong X. miR-488 inhibits cell growth and metastasis in renal cell carcinoma by targeting HMGN5. Onco Targets Ther. 2018;11:2205–2216. doi:10.2147/OTT.S156361. 29713189 PMC5912367

[39] Yu DL, Zhang T, Wu K, Li Y, Wang J, Chen J. MicroRNA-448 suppresses metastasis of pancreatic ductal adenocarcinoma through targeting JAK1/STAT3 pathway. Oncol Rep. 2017;38:1075–1082. doi:10.3892/or.2017.5781. 28677798

[40] Wang YB, Shi Q, Li G, Zheng JH, Lin J, Qiu W. MicroRNA-488 inhibits progression of colorectal cancer via inhibition of the mitogen-activated protein kinase pathway by targeting claudin-2. Am JPhysiol Cell Physiol. 2019;316:C33–C47. doi:10.1152/ajpcell.00047.2018. 30207785

[41] Malumbres M. Physiological relevance of cell cycle kinases. Physiol Rev. 2011;91:973–1007. doi:10.1152/physrev.00025.2010. 21742793

[42] Sherr CJ. Cancer cell cycles. Science. 1996;274:1672–1677. doi:10.1126/science.274.5293.1672. 8939849

[43] Al-Aynati MM, Radulovich N, Ho J, Tsao MS. Overexpression of G1-Scyclins and cyclin-dependent kinases during multistage human pancreatic duct cell carcinogenesis. Clin Cancer Res. 2004;10:6598–6605. doi:10.1158/1078-0432.CCR-04-0524. 15475449

[44] Malumbres M, Barbacid M. Cell cycle, CDKs and cancer: achanging paradigm. Nat Rev Cancer. 2009;9:153–166. doi:10.1038/nrc2602. 19238148

[45] Downward J. Targeting RAS signalling pathways in cancer therapy. Nat Rev Cancer. 2003;3:11–22. doi:10.1038/nrc969. 12509763

[46] Weinberg RA. The retinoblastoma protein and cell cycle control. Cell. 1995;81:323–330. doi:10.1016/0092-8674(95)90385-2. 7736585

[47] Wang W, Abbruzzese JL, Evans DB, Larry L, Cleary KR, Chiao PJ. The nuclear factor-kappa BRelA transcription factor is constitutively activated in human pancreatic adenocarcinoma cells. Clin Cancer Res. 1999;5:119–127. 9918209

