# Supplementary figures and images for: The NF-κB/miR-488/ERBB2 axis modulates pancreatic cancer cell malignancy and tumor growth through cell cycle signaling
Source: Cancer Biol Ther. 2022 Mar 28;23(1):294–309. doi: 10.1080/15384047.2022.2054257 (PMC8966990; doi:10.1080/15384047.2022.2054257)

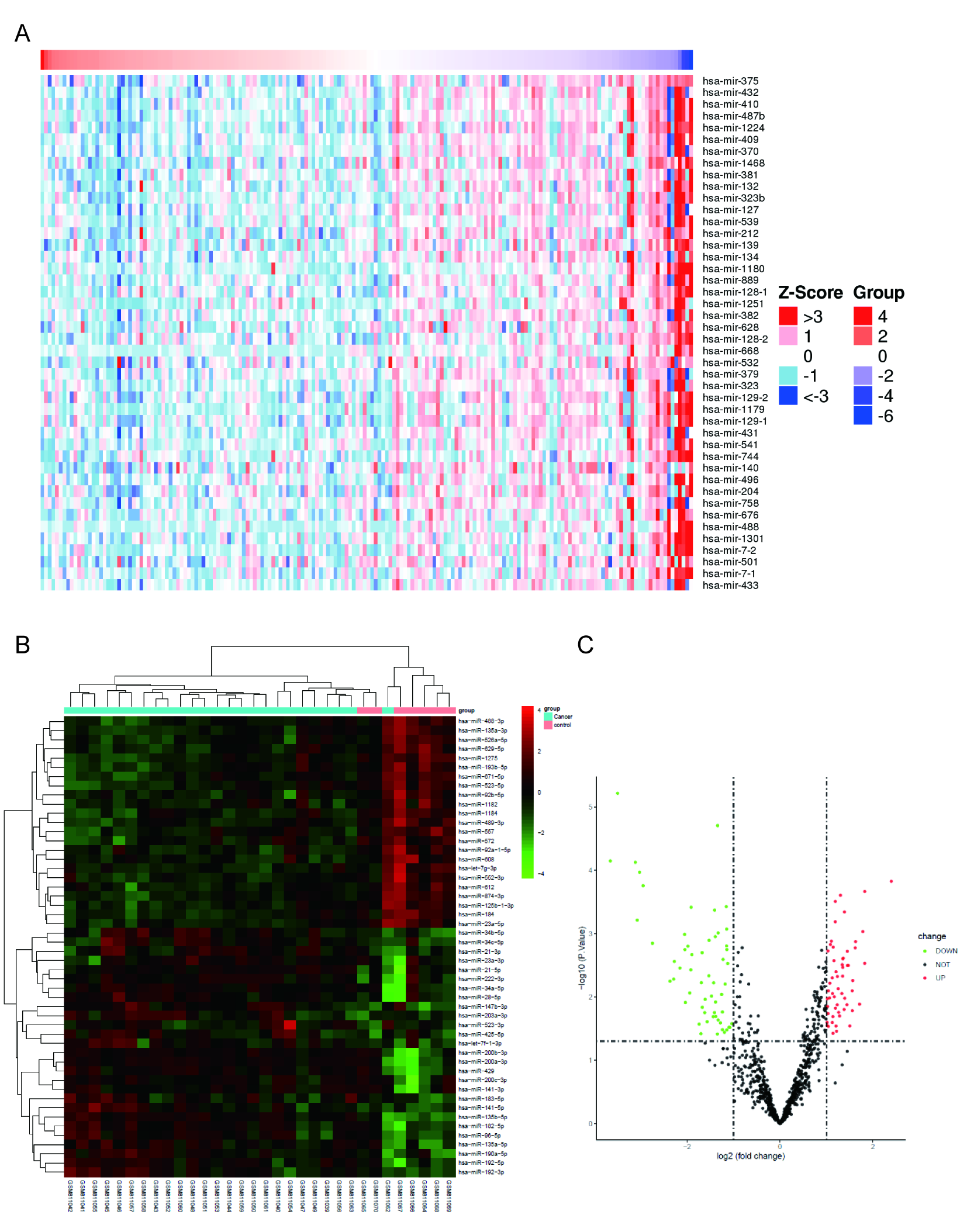

Supplement: Supplemental Material [file KCBT_A_2054257_SM2661.zip › fig.S1.tif]

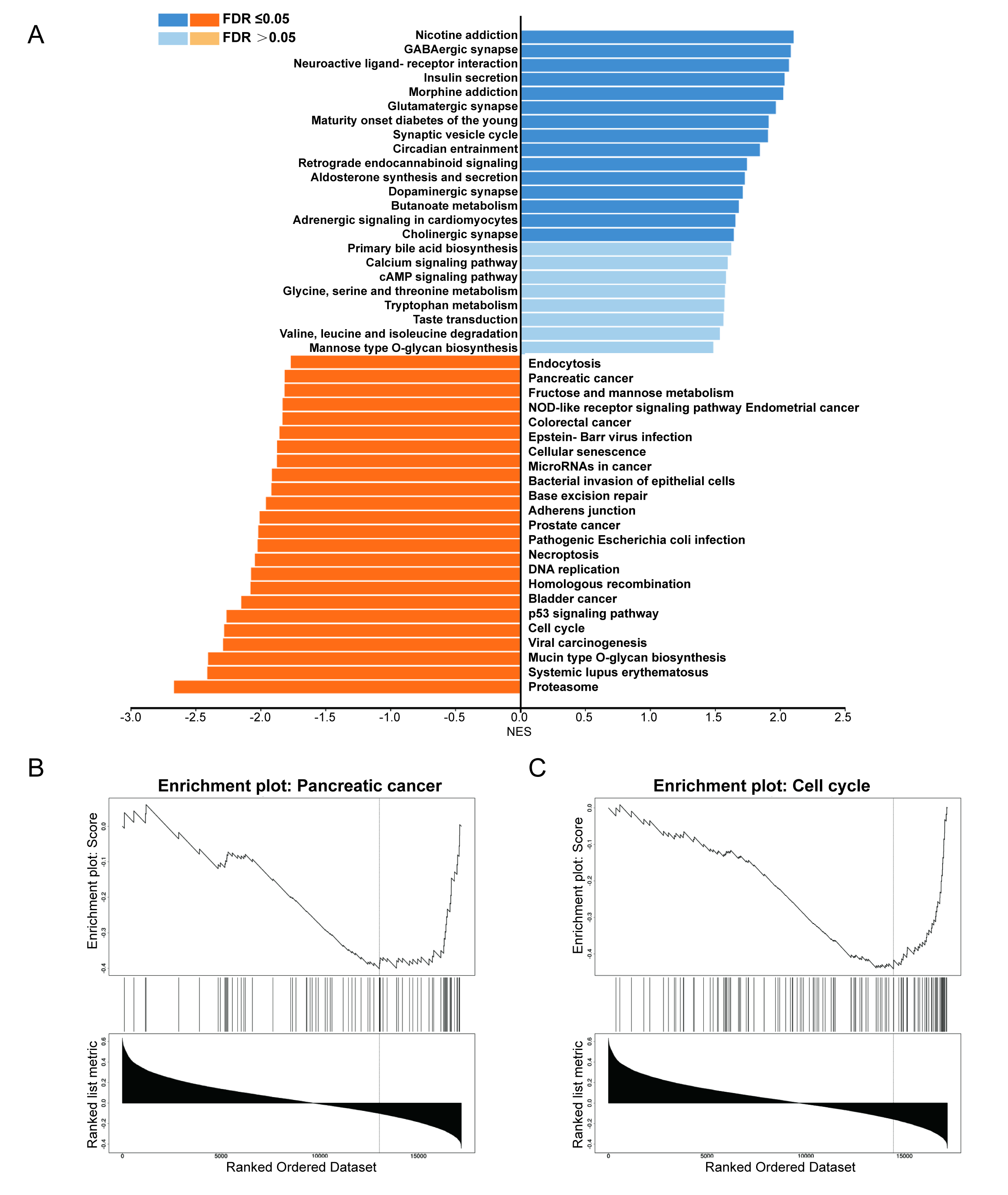

Supplement: Supplemental Material [file KCBT_A_2054257_SM2661.zip › fig.S2.tif]
